# Perceptions and Factors That Influence the Choice of Pathology as a Career Among Medical Students in Saudi Arabia

**DOI:** 10.7759/cureus.58094

**Published:** 2024-04-12

**Authors:** Abdulelah S Alharbi, Khalid A Alkhalifah, Omar A Alharbi, Mohammed T Alharbi, Jehad M Alabdulrahim, Majed Mohammed Wadi

**Affiliations:** 1 College of Medicine, Qassim University, Buraydah, SAU

**Keywords:** undergraduate medical student, student’s perception, pathology, career choice, factors

## Abstract

Introduction

Pathologists play a pivotal role in diagnosing diseases and improving patient care. Nonetheless, research indicates that a mere fraction of medical school graduates opt for a career in pathology, ranging from 1% to 3%. Diverse factors influence students' perceptions of pathology, encompassing lifestyle, patient interaction, and income.

Aim

The aim of this study was to assess the perceptions and factors that influence students' selection of pathology as a career in Saudi Arabia.

Methodology

A cross-sectional, self-administered electronic questionnaire was distributed among students from various regions of Saudi Arabia. The questionnaire encompassed both quantitative and qualitative data. To ensure statistical rigor, a confidence level of 95%, response distribution of 10%, and margin of error of 5% were applied. Accordingly, the recommended sample size of 150 participants was determined. Data analysis was performed using IBM SPSS Statistics for Windows, Version 24.0 (IBM Corp., Armonk, NY), with the chi-square test applied at a significance level of *P* < 0.05.

Results

Among the 664 participating students, 130 (19.6%) indicated an interest in pathology, of whom only 19 (2.9%) regarded it as their primary choice. No statistically significant difference was found between the male and female students or between the students in foundational and clinical years.

Conclusions

The female students generally held a more favorable perception of pathology, acknowledging its clinical significance and role in diagnosis, prognosis, and patient management. The absence of nocturnal calls emerged as the most encouraging factor, while limited patient interaction emerged as the primary deterrent in choosing pathology.

## Introduction

Deciding on a medical specialty is a pivotal moment for every medical student, as it shapes their professional trajectory. This decision, influenced by an array of factors, poses a formidable challenge given its enduring impact. Factors that affect the choice of medical specialty begin to exert their influence early, often stemming from initial interests sparked during their years in medical school. Exposure to specific specialties, personal encounters with various medical fields, mentorship, societal esteem, prestige, and lifestyle considerations all contribute to this intricate decision-making process [1,2]. Additionally, a study led by Ajaz et al. unveiled that certain medical fields, like psychiatry and general practice, are being avoided by students as potential career options due to negative feedback and comments, including those made in jest, from both peers and medical professionals, making them having certain stereotypes unwanted by the students [3].

Among the diverse medical specialties, clinical fields can be categorized into six groups: surgical, diagnostic, public health, psychiatry, anesthesiology and emergency medicine, and internal medicine [4]. Studies have consistently demonstrated a preference for surgical specialties among most medical students [5,6], which is potentially attributed to their relatively higher incomes [5].

Within the realm of diagnostic specialties lies the complex landscape of pathology, which includes more than 20 subspecialties [7]. Pathology intricately involves the analysis of patient specimens, tissue samples, and bodily fluids [8]. Leveraging specific tests and laboratory equipment, including microscopes and cutting-edge methodologies, pathology contributes decisively to diagnosis [7]. Its pivotal role extends across medical domains, with pathology-based tests constituting more than 70% of all diagnoses [8]. Clinical pathologists play crucial roles in diagnosing infectious diseases, diagnosis of neoplastic and nonneoplastic lymphoid and hematopoietic disorders, and pioneering genetic diagnostics for certain ailments, while anatomical pathologists play an important role in gross and microscopic analyses of tissues, examination, and diagnosis of single cells and small cell clusters taken from liquid specimens [7,8].

Despite pathology's far-reaching significance, it often remains overshadowed because of insufficient exposure during clinical years and rotations [9]. This lack of engagement results in limited comprehension of pathologists' roles, impact on patient management, lifestyle, and other pertinent considerations for career selection. Consequently, many students overlook pathology as a prospective field for their future endeavors [10]. International statistics have revealed that merely 1%-3% of medical school graduates from countries such as Australia, Canada, the United States, and the United Kingdom opt for a career in pathology [11-14]. In Saudi Arabia, studies conducted in Dammam and Riyadh have underscored similar trends, where only a minute fraction of medical students, ranging from 1.1% to 5%, express interest in pursuing pathology [10,15].

Multiple factors converge to explain this phenomenon, including limited exposure to the field, a lack of innate interest, minimal patient interactions, and comparatively lower-income prospects [16]. Given the undeniable impact of pathology on patients' lives, this study seeks to unravel the dynamics that motivate and dissuade medical students in Saudi Arabia from choosing a career in pathology. By identifying these factors, this research aimed to shed light on how to foster a deeper appreciation for this essential medical specialty.

This article was previously presented as a meeting abstract at the 9th Cultural and Scientific Week for Universities and Institutions of Higher Education in Gulf Cooperation Countries (GCC) on February 26, 2024.

## Materials and methods

Study design and population

A cross-sectional study design was used to investigate medical students’ perspectives across diverse regions of Saudi Arabia. The participants were recruited from various universities within the country by using a self-administered E-questionnaire disseminated through student groups.

Our populations in this study are medical students in both basic and clinical study phases. In the basic phase, students focus on building a foundation in medical sciences through classroom-based learning, lasting two to three years. During the clinical phase, students gain hands-on experience in hospital settings and real patient care scenarios, integrating theoretical knowledge with practical application over another two to three years.

We included in our study all undergraduate medical students who are studying at governmentally funded colleges of medicine in Saudi Arabia. Premedical year students, medical students at private universities, and medical interns were excluded from our study.

Sample size calculation

Determining the sample size factored in a 95% confidence level, 10% response distribution, and 5% margin of error. In light of these parameters, a suggested sample size of 150 participants was ascertained.

Study tool

For data collection, a self-administered E-questionnaire was meticulously crafted. Drawing from existing literature and expert consultations, including input from a pathologist, a medical educator, and medical students, the questionnaire's content validity, relevance, and appropriateness were evaluated.

The E-questionnaire consisted of four sections, comprising a total of 21 questions. The initial section gathered demographic data, including age, sex, nationality, study phase, university year, and cumulative grade point average (GPA). The second section prompted the students to rank their three most preferred future specialties post-medical school and identify the personal determinants that influenced their specialty selection. Similarly, the students indicated their three least preferred specialties. The third segment assessed the students' perceptions of the significance of pathology in clinical practice. Finally, the fourth section inquired about the student's perceptions of pathology as a career choice and elicited recommendations for enhancing interest in pathology as a future career.

Data collection

We enlisted data collectors, who are not considered co-authors; they are medical students from various universities across the Kingdom recruited through online channels to facilitate broad engagement with medical students in Saudi Arabia. Subsequently, each data collector was tasked with gathering data from their respective peers within the medical school community.

Data analysis

The collected data were analyzed using IBM SPSS Statistics for Windows, Version 24.0 (IBM Corp., Armonk, NY). Associations between the participants' demographic attributes and their inclination toward selecting pathology as a career were evaluated using the chi-square test. A *P*-value < 0.05 signified statistical significance, indicating a substantial association.

Ethical approval

Before data collection, ethical approval was granted by the Committee of Research Ethics of the Deanship of Scientific Research, Qassim University (No. 23-28-01). Rigorous scrutiny of the research protocol and procedures ensured their alignment with ethical standards, safeguarding participants' rights and well-being. Informed consent was obtained from all participants to guarantee their voluntary participation. Throughout the data collection and analysis, the confidentiality and anonymity of the participants' responses were stringently upheld.

## Results

A total of 644 participants were enrolled in the study, comprising medical students from various government universities across Saudi Arabia. Their data were collected using an E-questionnaire. Among the participants, 422 (63.6%) were female and 242 (36.4%) were male. A minor proportion (7, 1.1%) represented non-Saudi individuals. Most respondents hailed from Western (262, 39.5%) and Central (184, 27.7%) regional universities, followed by Eastern (125, 18.8%), Southern (78, 11.7%), and Northern (15, 2.3%) regional universities. In terms of the academic phase, 430 (64.8%) were in the clinical phase, while 234 (35.2%) were in the basic phase. The largest segment of participants were in their fourth year (235, 35.4%), with nearly half (326, 49.1%) maintaining a GPA between 4.51 and 5. For additional information, refer to Table 1.

**Table 1:** Demographic characteristics of the medical students. ^*^Study phases are basic and clinical. Basic phase: Initial years of medical education, emphasizing the establishment of a foundation in basic medical sciences. It primarily involves classroom-based learning without exposure to hospitals or direct patient interaction, typically lasting two to three years after the premedical year. Clinical phase: Final years of medical school, where students engage in hospital settings and apply their knowledge to real patient care scenarios. This phase, lasting two to three years, allows students to integrate theoretical knowledge with practical experience in medical practice.

Variable	Characteristics	Number	Percentage
Gender	Male	242	36.4%
	Female	422	63.6%
Nationality	Saudi	657	98.9%
	Non-Saudi	7	1.1%
Region	Central	184	27.7%
	Western	262	39.5%
	Eastern	125	18.8%
	Southern	78	11.7%
	Northern	15	2.3%
Phase of study*	Basic	234	35.2%
	Clinical	430	64.8%
Year of study	First	63	9.5%
	Second	115	17.3%
	Third	107	16.1%
	Fourth	235	35.4%
	Fifth	144	21.7%
GPA	4.51-5.0	326	49.1%
	4.01-4.5	196	29.5%
	3.51-4.0	88	13.3%
	3.01-3.5	41	6.2%
	<3.0	13	2%

**Figure 1:**
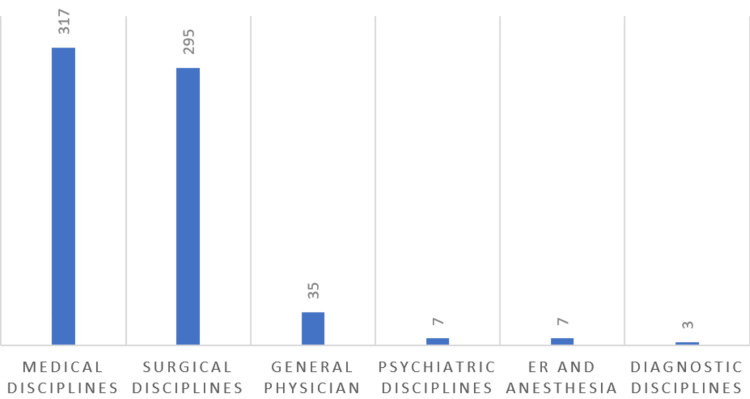
Preferred specialty of choice by medical students.

In terms of specialty preference, medical and surgical specialties emerged as the foremost choices, selected by 317 (47.7%) and 295 (44.4%) participants, respectively. Conversely, only three participants expressed an inclination for diagnostic specialties. When contemplating personal factors influencing specialty choice, 561 (85.4%) participants cited *personal interest*, while 48 (7.2%) attributed their selection to *My own experience during university*. Figure 1 and Table 2 offer further insights.

**Table 2:** Personal factors that influence specialty choice.

Factors	Frequency	Percentage
Personal interest	561	84.5%
My own experience during university	48	7.2%
Job opportunities	16	2.4%
Community’s bias toward more well-known specialties	11	1.7%
Role model	8	1.2%
Job environment	7	1.1%
Interaction with patients	3	0.5%
Level of stress	3	0.5%
Money	3	0.5%
Work-life balance	3	0.5%
Residency program (difficulty in matching and length of the program)	1	0.2%

To gauge students' comprehension of the importance of pathology, questions regarding its role in clinical practice, diagnosis, prognosis, and management were posed. The participants ranked the importance of pathology on a scale of 1 to 5, with 5 denoting high importance and 1 signifying low importance. Gender-based comparisons revealed that, overall, the female participants attributed statistically significantly greater importance to pathology in all aspects (Table 3). The mean importance rating was 4.21 for females and 3.92 for males in clinical practice (*P* = 0.004), 4.30 for females and 4.11 for males in diagnosis (*P* = 0.034), 4.19 for females and 3.84 for males in prognosis (*P* = 0.001), and 3.90 for females and 3.57 for males in management (*P* = 0.002).

**Table 3:** Importance of the pathology scale among the medical students. Statistical test: paired samples t-tests.

Question	Variable	Mean (SD)	Mean difference	Confidence interval	*P*-value
Do you think pathology is important clinically?	Male	3.92 (1.35)	−0.29	-0.49 to -0.09	0.004
	Female	4.21 (1.18)			
Do you think pathology has a role in diagnosis?	Male	4.11 (1.14)	−0.19	-0.37 to -0.02	0.034
	Female	4.30 (1.10)			
Do you think pathology has a role in prognosis?	Male	3.84 (1.23)	−0.35	-0.53 to 0.16	0.001
	Female	4.19 (1.14)			
Do you think pathology has a role in management?	Male	3.57 (1.34)	−0.33	-0.53 to 0.13	0.002
	Female	3.90 (1.25)			

**Figure 2:**
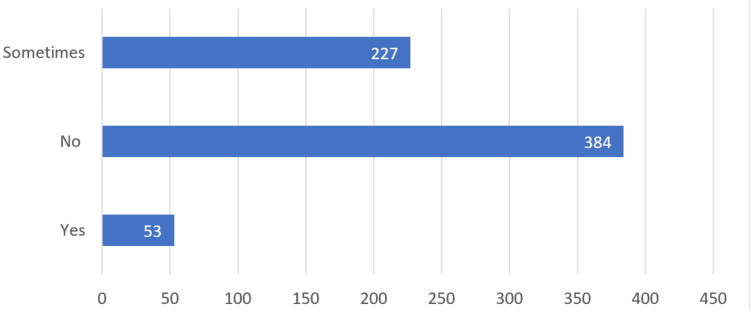
The bar chart illustrates medical students' perspectives on pathology (the question of whether pathology could be ignored in clinical practice).

**Figure 3:**
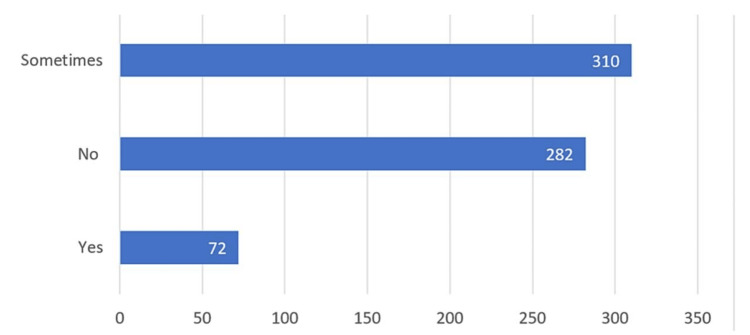
The bar chart illustrates medical students' viewpoints on pathology and whether other specialties could replace it in clinical practice.

When queried about the possibility of disregarding pathology in clinical practice, 384 (57.8%) participants responded *No*, 227 (34.2%) responded *Sometimes*, and 53 (8%) responded *Yes *(Figure 2). Similarly, on the question of whether other specialties could replace pathology in clinical practice, 310 (46.7%) participants responded *Sometimes*, 282 (42.5%) responded *No*, and 72 (10.8%) responded *Yes* (Figure 3).

In relation to pursuing pathology as a career, only 19.6% of the participants who expressed interest in pathology (*First choice* and *Top 5 choices*) considered it a potential career option. Among these participants, only 19 (2.9%) designated pathology as their first career choice, while 229 (34.5%) perceived it as a *less likely choice* and 305 (45.9%) did not include pathology in their plans (Table 4).

**Table 4:** Choice of pathology as a career.

	Frequency	Percentage
First choice	19	2.9%
Among the top 5 choices	111	16.7%
Less likely choice	229	34.5%
Not in my mind	305	45.9%

The analysis of the interest levels in pathology by gender and study phase, as shown in Table 5, revealed that neither gender (*P* = 0.253) nor study phase (*P* = 0.094) significantly influenced the level of interest.

**Table 5:** Level of interest in pathology based on gender and study phase. Statistical test: chi-square test.

Variable	Characteristics	Interested in pathology, *n* (%)	Not interested in pathology, *n* (%)	Total	*P*-value
Gender	Male	77 (18.2)	345 (81.8)	422 (100)	0.253
	Female	53 (21.9)	189 (78.1)	242 (100)	
Study phase	Basic	54 (23.1)	180 (76.9)	234 (100)	0.094
	Clinical	76 (17.7)	354 (82.3)	430 (100)	

The participants were further asked to identify the factors that encouraged them to consider pathology as a specialty. Approximately half (49.7%) of them cited a *better lifestyle*, while 23.2% indicated *No night calls*. However, 8 (1.2%) responders typed *Nothing*, indicating that there were no encouraging factors influencing their choice of pathology (Table 6).

**Table 6:** Factors that encouraged medical students to consider pathology as a specialty.

Factor	Number	Percentage
Better lifestyle	330	49.7%
No night calls	154	23.2%
Low communication with patients	60	9.0%
Challenging nature of clinical diagnosis	43	6.5%
Variety of cases	28	4.2%
Research opportunity	27	4.1%
Teaching opportunity	11	1.7%
Nothing	8	1.2%
Personal interest	2	0.3%
Interesting	1	0.2%

Conversely, in terms of the factors that discouraged them from selecting pathology, most participants (203, 30.6%) indicated *income*, while 173 (26.1%) highlighted *low communication with patients* (Table 7).

**Table 7:** Factors that discourage medical students from considering pathology as a specialty.

Factor	Number	Percentage
Income	203	30.6%
Low communication with patients	173	26.1%
Low exposure to clinical pathology during clinical years	73	11.0%
Highly complex cases	69	10.4%
Length of residency	62	9.3%
Does not allow part-time jobs	47	7.1%
Requires a lot of reading and updating	27	4.1%
No interest in pathology	8	1.2%
Boring routine	1	0.2%
Others	1	0.2%

Moreover, the participants provided recommendations in response to an open-ended question. Their responses were categorized into three main themes: enhancing awareness about the specialty (24 responses), improving educational approaches (19 responses), and bolstering job prospects and income (8 responses).

## Discussion

The primary aim of our study was to delve into the perceptions and determinants that shape medical students' choices regarding a career in pathology in Saudi Arabia. Our findings revealed that among the 664 participants, medical and surgical specialties garnered the highest preference, with 317 (47.7%) and 295 (44.4%) students selecting them, respectively. These results show a slight disparity from those of international studies conducted in countries such as Botswana [17], Syria [18], Sudan [19], United Arab Emirates [20], and Bahrain [21], where a greater leaning toward surgery was observed. This variance could be attributed to the fact that most Saudi Arabian universities' internal medicine course typically outlasts the general surgery course.

In terms of the factors that influence specialty selection as shown in Table 2, our respondents consistently cited personal interest and university experiences as crucial determinants. These findings mirror a parallel study in Riyadh [10]. Conversely, an Australian study [14] highlighted job environment and residency programs as the principal influencers of specialty preference. This discrepancy might have stemmed from the Saudi medical residency system, which is designed to maintain uniform teaching environments, learning experiences, and outcomes across different hospitals and programs.

Concerning pathology as a career choice, only 19 (2.9%) participants considered it their primary career option, whereas 111 (16.7%) included it in their top five choices. These numbers were lower than those reported in a multi-institutional study in Saudi Arabia [10], in which 5% of students designated pathology as their foremost preference. Similarly, a study at Imam Abdulrahman Bin Faisal University reported that only 0.7% of students aimed to pursue pathology after graduation [22]. A related study in Nigeria echoed this trend, where a paltry 2% of participants aspired to a career in pathology [23].

Various factors emerged as potential attractors or deterrents for medical students contemplating a pathology career. The students were drawn to the field by the promise of an improved lifestyle, and the lack of night calls ranked as another encouraging factor. These findings resonate with an Indian study that identified a stress-free lifestyle as a significant motivator for considering pathology as a profession [24]. Conversely, income disparities and limited patient interaction were the primary factors that discouraged students from pursuing pathology. This finding mirrors a study in the United States where medical students expressed apprehensions about the financial prospects for pathologists [25].

Our study found no significant disparity in pathology preference between the students in the basic and clinical phases, or between the male and female participants. However, the female students exhibited a markedly superior perception of pathology, acknowledging its clinical significance and role in diagnosis, prognosis, and patient management. A similar trend was observed in an Egyptian study where female students ranked clinical pathology as their second career choice [26]. In South Korea, a previous study suggested that women prioritize a lower workload and reduce the risk of occupational hazards, while men emphasize intellectual stimulation and prestige [27].

To enhance the perceptions of and interest in pathology, the participants highlighted the importance of increasing awareness about pathology as a career option. This deficiency in awareness appeared pervasive, as exemplified by a parallel study in Pakistan in which senior medical students expressed a desire for increased exposure to pathology during clinical years [16]. Furthermore, the participants advocated for more workshops, conferences, and opportunities to augment their understanding of pathology and its significance in clinical practice. To address these concerns, we suggest integrating visits to pathology laboratories during clinical rounds, fostering stronger interactions between students and pathologists, introducing pathology-focused conferences, and allocating dedicated time for pathology in the undergraduate curriculum. A Nigerian study suggested that an insufficiently taught pathology course during undergraduate years could contribute to a lack of interest in pursuing pathology as a future career [28]. In addition, introducing a post-sophomore program could be beneficial. A 2015 survey by the Program Directors Section of the Association of Pathology Chairs found that 47.7% of post-sophomore fellows applied for a pathology residency program [29].

Our study is distinguished by its substantial cohort of medical students representing diverse universities spanning various regions of Saudi Arabia, encompassing both foundational and clinical phases of their education. Additionally, we meticulously incorporated student feedback in our survey to optimize their engagement with pathology, while identifying and addressing impediments encountered during their college tenure. This endeavor is deemed crucial for fostering informed decision-making regarding pursuits in pathology among students.

While our study contributes valuable insights, acknowledging its limitations is crucial. First, internship students were not included, which could have offered diverse perspectives. Second, the study was confined to government universities in Saudi Arabia, potentially limiting its generalizability. Including nongovernment universities could offer a broader view of medical students' interests. Third, the underrepresentation of participants from the northern region suggests the need for further exploration in that area. Finally, reliance on an E-questionnaire could introduce certain biases such as selection bias and acquiescence bias.

## Conclusions

Our study underscores that only 19.6% of the medical students expressed interest in pathology, with only 2.9% considering it their primary career choice. Remarkably, neither gender nor academic phase significantly influenced their interest levels, although the female students displayed a better grasp of the clinical relevance of pathology. The allure of an improved lifestyle was enticing, while income concerns were deterrents. Increasing awareness and addressing factors that influence interest in pathology could bridge this gap. To gain a more comprehensive perspective, future research should extend to medical students in nongovernment universities, students in northern regions, and internship medical students.
